# Erythropoiesis In Vitro—A Research and Therapeutic Tool in Thalassemia

**DOI:** 10.3390/jcm8122124

**Published:** 2019-12-02

**Authors:** Eitan Fibach

**Affiliations:** The Hematology Department, Hadassah—Hebrew University Medical Center, Jerusalem 91120, Israel; Fibach@yahoo.com; Tel.: +792-2-6776751; Fax: +792-2-6423067

**Keywords:** thalassemia, erythroid cells, cultures, hemoglobin

## Abstract

Thalassemia (thal) is a hereditary chronic hemolytic anemia due to a partial or complete deficiency in the production of globin chains, in most cases, α or β, which compose, together with the iron-containing porphyrins (hemes), the hemoglobin molecules in red blood cells (RBC). The major clinical symptom of β-thal is severe chronic anemia—a decrease in RBC number and their hemoglobin content. In spite of the improvement in therapy, thal still severely affects the quality of life of the patients and their families and imposes a substantial financial burden on the community. These considerations position β-thal, among other hemoglobinopathies, as a major health and social problem that deserves increased efforts in research and its clinical application. These efforts are based on clinical studies, experiments in animal models and the use of erythroid cells grown in culture. The latter include immortal cell lines and cultures initiated by erythroid progenitor and stem cells derived from the blood and RBC producing (erythropoietic) sites of normal and thal donors, embryonic stem cells, and recently, "induced pluripotent stem cells" generated by manipulation of differentiated somatic cells. The present review summarizes the use of erythroid cultures, their technological aspects and their contribution to the research and its clinical application in thal. The former includes deciphering of the normal and pathological biology of the erythroid cell development, and the latter—their role in developing innovative therapeutics—drugs and methods of gene therapy, as well as providing an alternative source of RBC that may complement or substitute blood transfusions.

## 1. Introduction

### Thalassemia

Thalassemia (thal) is an autosomal recessive hereditary hemolytic anemia because of a partial or complete deficiency in the synthesis of one of the globin chains, mainly the α (α-thal) or β (β-thal), which compose, together with the iron-containing protoporphyrin—heme, the major adult hemoglobin (HbA), a tetramer of α_2_β_2_. It is caused by one or more of several hundred mutations in the corresponding genes [1].

The major clinical symptom of β-thal is chronic anemia—a reduced number of RBC and their Hb content, resulting from a deficiency in Hb production and increase destruction of mature RBC in the circulation and their precursors in the bone marrow (BM) (hemolysis). The anemia incapacitates the oxygen-carrying capacity of the blood—leading to hypoxia throughout the body. The anemia and other symptoms in β-thal is due mainly to oxidative stress, a state of imbalance between oxidants and antioxidants. Although oxidative stress is not the primary etiology of thal, it mediates several abnormalities in erythroid cells and other cells throughout the body. Excess oxidants, such as the reactive oxygen species (ROS), interact with various cellular components, such as the DNA, proteins, and membrane lipids, resulting in cytotoxicity and vital organ (e.g., heart, liver) failure. The oxidative stress in β-thal is due to: (A) The toxic effects of the unpaired α-globin chains, which are unstable; they precipitate intracellularly as hemichromes that bind to the cell membrane. (B) Excess of iron (iron overload) due to recurrent blood transfusions, the standard treatment of the chronic, severe, anemia of patients with intermediate/major thal) and augmented absorption of nutritional iron. Free (unbound) iron catalyzes the Fenton reaction that generates excess ROS [2].

Currently, thanks to the significant improvement in therapy, mainly regular blood transfusions and administration of iron-chelator agents, as well as, when appropriate and available, allogeneic hematopoietic stem cell transplantation, the morbidity and mortality of patients with β-thal is decreased. However, the advanced age of the patients and the length of the treatment generate new symptoms. Examples of the latter are the consequences of RBC transfusions, which in severe cases, are performed every 3 weeks. This affects the patient’s quality of life, may cause recurrent infections and immune reactions, and, above all, iron overload—the major cause of morbidity and mortality, especially among elderly patients [3,4,5].

Although prevention strategies, mainly by prenatal diagnosis, have been implemented in many centers, thal is still the most common monogenic inherited disease worldwide. It originated and spread around the Mediterranean, the Middle East, and Southeast Asia, coincidental with the occurrence of malaria (carriers of the thal, as well as the sickle cell anemia, genes are considered to be resistant to the malaria parasite) [6]. Today, due to vast immigration, thal patients are present around the globe [7] and their incidence increases steadily.

Thal severely affects the quality of life of the patients and their families and imposes a substantial financial burden on the community (especially in low-income countries). These considerations position β-thal, among other hemoglobinopathies such as sickle cell disease, as major health and social problem that deserves increased efforts in research and its clinical application.

The study of the pathophysiology of thal and the development of new therapeutic modalities that have been based primarily on clinical studies of the patients have been aided by preclinical studies using in vivo and in vitro experimental systems. Thal is not known to occur naturally in animals, but molecular manipulations of mice have generated thal models [8]. In vitro studies have been accomplished also in cultures of erythroid cells derived from normal individuals and patients. The present review summarizes the use of erythroid cultures, their technological aspects and their contribution as research and therapeutic tools. The former includes deciphering of normal and pathological biology of erythroid cell development, and the latter involves their potential use as a source of RBC for transfusions and their role in gene therapy.

## 2. Erythropoiesis—In Vivo

RBC production (erythropoiesis) occurs normally in the BM as pluripotent hematopoietic stem cells (HSC) give rise to all blood cell lineages, including RBC (for review see [9]). During the development of the erythroid lineage, the HSC, through a series of stages, proliferate and differentiate into erythroid committed progenitors (EPC) that in turn give rise to more differentiation-advanced and morphologically-distinguishable, precursors—the basophilic, polychromatophilic, and orthochromatic erythroblasts. This process, which includes decreasing cell volume, nuclear condensation, and Hb production, is mediated and regulated by various cell–cell and cell–extracellular matrix interactions along with different cytokines, most notably, erythropoietin (EPO). The latter binds to a specific surface receptor (EPO-receptor) on EPC, leading to transmission of a signal that supports their survival, proliferation, and maturation [10]. Eventually, the orthochromatic erythroblasts extrude their nucleus and egress from the BM into the peripheral blood (PB) as reticulocytes. The reticulocytes, lose their remaining cellular organelles such as RNA and ribosomes and some plasma membrane, they shrink in size and acquire the biconcave disc shape to become, within 1–2 days, mature RBC (erythrocytes). RBC remain in the circulation for approximately 120 days before being cleared by macrophages in the reticuloendothelial system of the spleen and the liver.

The chronic anemia in thal and its resultant hypoxia result in overproduction of EPO, the main erythropoietic stimulating hormone, which stimulates increased production of RBC (chronic stress erythropoiesis). Other stimulating factors include members of the transforming growth factor-β and activin receptor-II trap ligands [11]. However, this compensatory attempt is futile (“ineffective erythropoiesis”) due to oxidative stress-induced apoptosis and abortive differentiation.

## 3. Erythropoiesis—In Vitro

### 3.1. Erythroid Cells Lines

Erythropoiesis has been studied in vivo in animal models and in human subjects, both normal and patients with diseases associated with the erythropoietic system. In-vitro, it has been studied using mainly two culture systems: Established, immortalized cell lines that can be induced to express some aspects of the erythroid phenotype, and primary cultures of EPC present in the BM or the PB. Examples of the former are the murine erythroleukemia cell line, which was established by C. Friend from the spleen of a virus-infected mouse [12], and the human K562 cell line, established by Lozzio C.B and Lozzio B.B from the PB of a patient with chronic myeloid leukemia in blast crisis [13]. These cells grow continuously suspended in liquid culture as undifferentiated erythroid progenitors as manifested morphologically, immunologically, and biochemically. Their leukemogenicity was demonstrated by injection into suitable experimental animals. When appropriately stimulated by drugs such as dimethylsulfoxide, hexamethylene bisacetamide, or butyric acid, they undergo terminal differentiation [4,14]. This differentiation is similar to normal erythropoiesis, including typical morphological changes, accumulation of globin mRNAs, synthesis of globin chains and heme, the formation of Hb, the appearance of RBC-specific membrane antigens, and cessation of cell division. These cell lines provide convenient experimental models and have been utilized to unravel important aspects of erythroid development, such as the production of globin mRNA [15]. Their establishment is, however, a rare, random event and they and they lack some aspects of normal erythropoiesis, such as the responsiveness to EPO.

### 3.2. Erythroid Cultures Derived from Progenitor Cells

Primary erythroid cultures involve the growth of HSC or progenitor cells in vitro for relatively short-term (several weeks) periods. The studies, pioneered about 50 years ago, independently, in the laboratories of Leo Sachs [16] and David Metcalf [17], involved the seeding of BM cells in semi-solid agar-containing medium on top of a feeder layer. Under these conditions, most of the cells disintegrated within a few days, but some cells, termed colony-forming units (CFU), produced discrete colonies of granulocytes, monocytes and macrophages [18]. Colony formation required the presence of glycoproteins, termed colony-stimulating factors (CSF), produced and secreted by the feeder cells, which supported the proliferation and differentiation of myelomonocytic progenitors [18]. The feeder was subsequently substituted for conditioned medium–spent medium of cultures of various cell types. These culture provided crucial bioassays for the purification and characterization of the granulocyte, macrophage, and granulocyte–macrophage CSFs and cloning of their genes.

Several years later, EPC were also demonstrated to form colonies in semi-solid medium [19]. In these cultures, the agar was replaced by methylcellulose or plasma clot, and the CSFs—by EPO. When human BM cells are cloned in these media, two types of colonies are produced: Small clusters of Hb-containing cells that develop 7 days after seeding and disappear later on to be replaced by large colonies composed of several clusters containing hundreds to thousands of Hb-containing cells that fully develop after 14 days. The two types of colonies are derived from EPC belonging to two developmental stages: The more mature EPC, termed erythroid colony-forming units (CFUe), produce the early-appearing, small, colonies, while the less mature EPC, termed burst forming units (BFUe), produce the late-appearing large colonies (bursts). In mice, the kinetics of colony development is faster; CFUe-derived colonies develop after 3–5 days while BFUe-derived colonies—after 7–10 days.

In the presence of a combination of cytokines, containing stem cell factor, granulocyte–macrophage CSF, EPO, and thrombopoietin [20], some colonies contain a mixed population of monocytes/granulocytes, erythroid cells, and platelet precursors. The initiator cell of such colony, termed CFU_GEMM_, was considered to represent in vitro HPC [21].

Cells obtained from individual colonies or collected from the whole plate are assessed for their numbers, differentiation state (by immune-phenotyping), as well as morphologically (by microscopic examination). Using this procedure, it is possible to study the frequency and nature of the EPC in various sources—the PB, BM, extra-medullary sites such as the spleen and the liver, the umbilical cord blood of the newborn and embryonic erythropoietic sites.

Cloning of cells in semi-solid medium has several advantages: Since each colony represents a clone, the number of colonies, their size and duration of development provide data on the frequency of specific EPCs in the tissue tested. However, the fact that the cells are immobilized in semi-solid medium has several disadvantages as well: (I) The yield is low (<10^6^ cell/mL culture), limiting the study of the growth kinetics as well as the biochemical, molecular, and immunological phenotype of the developing cells. (II) It is a one-step continuous culture, making it hard to add or withdraw components (i.e., EPO, CSF) to/from the culture in order to determine their effects on different maturation stages. (III) Cell–cell interactions are limited.

These disadvantages can be overcome in liquid suspension cultures. We have described such a culture that supports the growth of human EPC [22]. The procedure is separated into two phases: In the first phase, PB mononuclear cells, separated by density gradient centrifugation, are cultured for 1 week in alpha medium supplemented with fetal bovine serum, a combination of growth factors, provided as conditioned medium of the 5637 human bladder carcinoma cells or recombinant-derived cytokines, but in the absence of EPO. Cyclosporin A is added to prevent the activation and proliferation of lymphocytes. During this phase, BFUe, which are present in a small number in the initiating cell population, proliferate and differentiate into CFUe. In the absence of EPO, the development of the EPO-dependent CFUe is blocked, and they are accumulated and synchronized. In the second phase, the non-adherent cells are harvested, washed, and recultured in liquid medium containing fetal bovine serum, albumin, EPO, stem cell factor, the steroid hormone dexamethasone and the reducing agent β-mercaptoethanol. Under these conditions, the CFUe proliferate and mature, within 1 week, into proerythroblasts. Other types of progenitors disappear due to the absence of their growth factors. At this stage, a purification step on Percoll density gradient can be included. The proerythroblasts are much larger and less dense than the other cells present, mainly residual lymphocytes and RBC. The separated proerythroblasts, re-cultured in the same medium, mature within an additional week into Hb-containing nucleated orthochromatic normoblasts. This culture procedure yields large and relatively pure and synchronized (in terms of differentiation) erythroid populations [23]. Since the erythroid cells grow in suspension, aliquots can be sampled at several times without disturbing the cultures, and assayed for various parameters, such as morphology, size, number, viability, apoptosis, cell cycle, surface antigens, and gene expression. Since peripheral blood mononuclear cells (fractionated by Ficoll separation), rather than purified EPC, initiate the culture, the procedure is relatively simple and non-expensive. Nevertheless, since EPC eventually undergo terminal differentiation into non-dividing cells, their expansion potential is limited.

Many reports have described modifications and improvements of this two-phase liquid culture protocol. These included the use of defined cytokine cocktails, serum-free medium [24,25] and feeder layers. For example, the use of mouse bone marrow stromal cells (MS-5) in the last phase of the culture protocol facilitated the final maturation, producing nearly 100% enucleated RBC. Subsequent studies reported a high rate of enucleation without feeder layers [26]. Steroids, such as dexamethasone, were found to induce extensive proliferation of early EPC [27]. The Migliaccio lab has developed a simplified procedure, they termed human erythroid massive amplification (HEMA) culture, to expand human blood mononucleated cells and has shown, using a variety of in vitro assays, that human RBC produced in the presence of high-levels of steroids were highly similar to their in vivo-produced counterparts [28].

### 3.3. Erythroid Cultures Derived from Stem Cells

As mentioned above, EPC have limited expansion and differentiation potentials. In contrast, HSC possess self-renewal, proliferation as well as pluripotent differentiation capabilities. Self-renewal into daughter stem cells permits them to maintain, or increase, their pool, depending on needs, while proliferation and differentiation are responsible for the production of mature cells. HSC obtained from murine bone marrow were first expanded in long-term cultures on adherent bone marrow-derived feeder layer by Dexter et al. [29].

Human stem cell cultures were originated from various sources, such as embryonic, neonatal cord blood, bone marrow, and peripheral blood as well as induced pluripotent stem cells (iPSC) derived from somatic cells. Although theoretically, these cells can be maintained by self-renewal indefinitely, a property that makes them ideal founder of continuous erythroid cultures, in vitro their self-renewal is limited by their tendency to undergo terminal differentiation into non-cycling cells. To inhibit HSC differentiation, we have employed various strategies, including addition of the copper chelator tetraethylenepentaamide [30], the retinoic acid receptor antagonist AGN 194310 [31,32] or nicotinamide [33].

Human embryonic stem cells (hESC) were reported to proliferate and differentiate into erythroid cells by co-culturing on a feeder layer of a murine BM cell line or a yolk sac endothelial cell line [34]. The Bouhassira lab expanded on these studies by co-culturing hESs with human fetal liver cells, showing that their in vitro differentiation closely paralleled their development in vivo—they sequentially produced cells containing embryonic and fetal Hbs [35]. Similar findings were reported by other labs using a variety of methods to increase the yield of erythroid cells from hESCs. The use of hESC, however, is of limited availability and it arouses ethical and religious objections.

Neonatal cord blood (CB) is a rich source of HSC, which are often collected and freeze-stored for HSC transplantation. Because of the limited volume of the collected CB, the number of its HSC is sufficient for treating children but not adults. One way to overcome this problem is to transplant HSC pooled from several CB units, or to expand CB HSC in vitro. For example, Zhang et al. [36] have developed a procedure based on a cytokine cocktail (stem cell factor, thrombopoietin, insulin-like growth factor 2, fibroblast growth factor 2, and angiopoietin-like proteins) to amplify HSC about 20 fold [36]. More recently, Boitano et al. expanded HSC by 50-fold using SR-1, an aryl hydrocarbon receptor antagonist [37].

The PB contains a relatively small number of HSC, but it is available from normal donors and patients, including thal patients. A larger number of blood-derived HSC can be obtained from unused mobilized HSC and from leuko-reduction byproducts (buffy coats) of blood transfusion units.

The HSC culture protocol usually starts by immune-purification of these cells based on their surface antigens (e.g., CD34, CD133). The culture methodologies vary widely but usually involve three phases of liquid cultures. The cultures are supplemented with reagents that promote expansion, commitment and maturation to erythroid cells.

Recently, induced pluripotent stem cells (iPSCs), produced by molecular manipulation of somatic cells have been studied as potential sources of HSC-derived erythroid cultures. For example, the Douay lab used iPSC lines obtained from human fetal and adult fibroblasts. The protocol comprised two steps: Differentiation—by formation of embryoid bodies to obtain EPC, and maturation to enucleated, fetal Hb (HbF)-containing RBC, using cytokines and human plasma-containing medium. The protocol avoided the passage through a hematopoietic progenitor, a cellular stroma feeder layer and use of proteins of animal origin [38].

## 4. The Use of Erythroid Cultures for Research and Diagnosis

Although erythroid cultures do not precisely mimic the in vivo conditions, they, recapitulate many aspects of in vivo erythropoiesis. As such, they have been utilized for basic research of erythroid cell development, for devising novel therapeutic modalities and, in some cases, for determining the diagnosis and prognosis. For example, the “two-phase liquid system” [22] has been utilized to study normal and pathological erythroid cell development. This included, among others, the analysis of the cell cycle and growth kinetics induced by EPO [39], the expression of the ABH antigens [40], band 3 deficiency in patients with hereditary spherocytosis [41], the effects of antioxidants [42] and iron-chelating agents [43], and externalization and shedding of phosphatidylserine [44]. It was also used for testing novel HbF inducers [45], for validation of gene therapy strategies based on novel lentiviral vectors [46,47], for gene editing [48,49].

### 4.1. Clinical Diagnosis

Primary cultures have been utilized in some cases for clinical diagnostic. Examples include red cell aplasia, where EPC fail to grow in the presence of EPO [50], and polycythemia vera where they can grow in the absence of EPO [51]. The assay of the proliferation/differentiation ability of EPC carried out in semi-solid (endogenous colonies) or liquid cultures [52,53] used to be a standard diagnostic test for polycythemia vera. It has been largely replaced by molecular analysis of genes encoding proteins involved in the EPO signaling pathway, particularly Jak-2 [54]. However, primary cultures can still help diagnose patients with myeloproliferative disorders with normal (wild type) Jak-2 gene. Another clinical aspect that can be studied in vitro is the presence of erythropoiesis inhibitors, either cellular or humeral, in patients’ blood [55].

In thal, the number of EPC and their ability to develop in culture is much increased, especially in splenectomized patients [56]. There is a positive correlation between the number of colony-developing EPC and the number of circulating nucleated RBC (normoblasts). The spleen acts as a filter for circulating immature cells. In thal, the chronic anemia stimulates erythropoiesis, and more EPC and normoblasts enter the circulation, especially following splenectomy that abolishes the filtering effect of the spleen.

### 4.2. Bio-Banking of Erythroid Cells

The clinical presentation of thal is diverse, depending on the genetics, epigenetics, and many other parameters, of individual patients. Each patient, therefore, has a personalized schedule of treatment, such as blood transfusion and iron chelation. Thus, a single-center research, and especially multi-center collaborative network research is seriously limited by the patients’ recruitment and sample collection. Bio-banking is a relatively new and important solution [57,58]. Such banks contain samples of body fluids, cellular components (such as DNA), whole tissues, and fresh or cultured cells derived from normal individuals or patients. Cells are collected, frozen and stored in the banks. Upon thawing, the samples are used either directly or following growth in culture. For the study of β-thal, bio-banking is of special interest. A validated cellular biobank for β-thal cells has been recently reported [59]. The procedure includes cellular in vitro expansion, cryopreservation, storage in liquid nitrogen and, finally, thawing and sub-culturing. Peripheral blood samples (25 mL) were drawn from patients prior to blood transfusion. Mononuclear cells were separated by Ficoll-Hypaque density gradient centrifugation and CD34^+^ cells—by immune-magnetic microbeads. The CD34^+^ cells were maintained in culture using two protocols: The two-phase liquid culture, as described above, and a serum-free medium supplemented with a “Cytokine Cocktail for Expansion of Human Hematopoietic Cells (Stem Cell Technologies, Vancouver, Canada), EPO and dexamethasone. Cell concentration was maintained below 5 × 10^5^/mL by their dilution with fresh medium. Once the cells reached maximal growth (between 7 and 12 days), they were frozen in vials of 5 × 10^6^ cells each and stored in liquid nitrogen. After thawing, the cells were washed and cultured in the same medium.

Studying identical cryopreserved samples in different labs produced similar results, indicating that the frozen cells retained their original pattern of maturation, including Hb production. This approach might provide an important biological source, facilitating the use of in vitro erythropoiesis for multiple goals. Among these is stratification of patients concerning the response to therapies, taking into account all the phenotypic/genotypic characteristics, and designing optimal treatments (e.g., for stimulation of HbF, iron chelation) targeting various symptoms at the individual patient’s level (personalized medicine). Finally, the Biobank might provide a cell template for gene manipulation of HSC or iPSC for each patient [60].

## 5. Developing Novel Therapeutic Modalities

### 5.1. Stimulation of HbF Production

During human prenatal life, HbF, a tetramer of α- and γ-globin (α_2_γ_2_), is the major Hb. It is replaced perinatally by HbA (α_2_β_2_) (Hb switching). Thus, the clinical features of β-hemoglobinopathies, including β-thal, are not apparent at birth; only as HbF levels wane the symptoms are manifested [61]. Patients with β-thal produce high, but variable, levels of HbF compared to normal individuals. High levels of HbF ameliorate the severity of the disease, mainly by reducing the surplus of α-globin chains [62].

These findings have motivated the research of Hb switching, and pharmacological and molecular approaches to reactivate the expression of the γ-globin genes and the production of HbF [63]. Primary erythroid cultures recapitulate the pattern of Hb production in vivo [64]—cultures derived from embryonic or fetal sources produce mainly the embryonic (Gower 1 and 2) and fetal Hbs, while cultures derived from adult sources produce mainly HbA. Erythroid cultures derived from thal patients were instrumental for studying the Hb switch and for screening for HbF-stimulating drugs [45]. For example, we demonstrated that the addition of hydroxyurea to such cultures significantly increased their HbF production [65]. This was demonstrated by measuring of HbF-containing cells (F-cells) by flow cytometry, of globin mRNA by quantitative PCR and of globin and HbF by a high-pressure liquid chromatography [66]. Using primary cultures, as well as the K562 cell line and monkey’s models yielded a long list of compounds with HbF-stimulating potential [67]. Currently, hydroxyurea is the only compound in routine clinical use [68]; but several drawbacks remain; being an S-phase cell cycle inhibitor, it is myelo-suppressive necessitating careful monitoring of the patients. In addition, its mechanism of action on HbF stimulation remains elusive, a subset of the patients is resistant, its effect in β-thal is inferior compared to that in sickle cell disease, and it is possibly teratogenic and carcinogenic. The research of other, more innocent and effective, drugs continues. New agents include those that affect chromatin regulators, such as decitabine on DNA methylation and histone deacetylase inhibitors and others that affect DNA-binding transcription factors [63].

Another point of interest, that has not been fully explored, is the use of primary cultures derived from the peripheral blood of patients prior to treatment might provide a predictive bioassay for response and thereby assist in selecting the optimal treatment.

### 5.2. Gene Therapy

Gene therapy involves molecular manipulation of the autologous HSC, which are then returned to the patient for reconstitution [69,70]. For β-thal, this approach has targeted the following: (A) Increasing the production of γ-globin by the addition of its gene, over-expression of its endogenous activating transcription factors and silencing of its repressors. (B) Increasing the production of β-globin by the addition of a normal gene or correction of the mutated gene. (C) Reducing the production of α-globin, thus decreasing its excess in β-thal patients. These gene manipulations have been carried out mainly with lentivirus vectors in several experimental systems, including cultured HSC from thal patients and in β-thal mouse models. Yet, the safety profile of such technologies is still uncertain.

Erythroid cultures derived from β-thal progenitors may assist the field of gene therapy at several levels. (I) They serve as an experimental model where the effectiveness of the gene modification procedures can be evaluated by analyzing (for their β- or γ-globin) the differentiated progeny of gene-modifying stem cells. (II) They permit HSC expansion providing an increased number of HSC before and after the gene manipulation procedure. (III) They may provide a potential selective environment for the enrichment of the modified cells.

## 6. Production of RBC for Transfusion

The main symptom of β-thal, chronic anemia, is treated by repeated blood transfusions. Patients with severe anemia (thal major) usually require 1–2 units of blood per month. Although blood supply is usually sufficient, occasional shortage may emerge. In developing countries, donor consent is low and the blood collection system is inadequate. In developed countries, future supply is expected to shrink due to a demographic trend of low birthrate, leading to an increased proportion of older people needing transfusions (e.g., patients with myelodysplastic syndromes) and smaller proportion of younger donors [71]. Also, chronically transfused patients, such as in thal, often develop serum antibodies directed against foreign donor RBC (allo-immunization) [72]. All these problems limit the availability of appropriate donor blood, particularly for those having rare blood types [73]. In addition, the risks of infection through transfusion always persist, particularly in countries where regular screening of donated blood for transfusion-transmissible infections, which include HIV and hepatitis B and C viruses, is not routinely performed [74,75]. For these reasons, additional sources of cells for transfusion are needed. The in vitro production of RBC in culture has recently emerged as a potential alternative.

A single blood unit contains about 2 × 10^12^ RBC. Although various protocols have been developed for producing cultured RBC (cRBC), achieving this large number has so far proven difficult and expensive. Production optimization is underway and the use of cRBC has recently entered clinical evaluation.

The ideal cells for establishing such cultures source should be widely availability (preferentially as discarded material) and have the potential to undergo extensive expansion and complete maturation into functional RBC. Potential sources are leukoreduction byproducts (buffy coats) of blood donations, neonatal CB samples that are insufficient for HSC transplantation, and unused PB mobilized HPC cells harvested for transplantation.

Recently, continuous production of cRBC, making use of established EPC lines derived from ESC or iPSC established by epigenetic/genetic manipulations, has been envisioned. Such cell lines would be equivalent to the megakaryocytic cell lines recently derived from human ESC capable to generate massive numbers of platelets in vitro [76,77]. Another interesting possibility allowed by advanced technology is to reprogram somatic cells directly into erythroblasts, bypassing the pluripotent state, by over-expression of suitable genes. This approach was demonstrated by over-expression of p45NF-E2/ Maf that turned human fibroblasts into megakaryocytes [78]. Another source of cRBC may come from erythroid cells endowed with high proliferative ability. Recently, a population of self-renewing erythroblasts has been discovered in early mouse embryos, and it was shown to possess self-renewal and extensive proliferative capacity in vitro [79].

To qualify for transfusion, the characteristics of the cRBC should match that of the in vivo produced RBC, especially concerning the following demands:

(A) The cRBC should not be immunogenic. Although cRBC express the same major blood group antigens as their in vivo counterparts [80], genetic changes that may occur during culture could affect their antigenic profile. Also, they may acquire neoantigens derived from proteins present in the culture media that adhere to their surface. Their antigenicity, therefore, should be carefully tested prior to transfusion. 

(B) The size of the cRBC and the deformability of their membrane should allow them to squeeze through the narrow capillaries and venules. The size of cRBC is similar, although slightly macrocytic, compared to that of in vivo-generated RBC.

(C) The oxygen-carrying capacity, which is determined mainly by the Hb type and content, of cRBC should match that of in vivo produced RBC. Although α-globin is preferentially expressed in immature erythroblasts, albeit with high donor-to-donor heterogeneity [81], the α/(γ+β) globin ratio nearly normalized during maturation probably due to high activity of the alpha hemoglobin stabilizing protein [82].

(D) Enucleation is important for the survival of RBC in the circulation. The presence of nucleated RBC, normoblasts, in the PB of splenectomized thal patients [1] suggests that the spleen, when present, removes these cells quite efficiently. In vivo, the enucleation process is facilitated by interactions with macrophages [83] in specific niches—“the erythroblast islands", of the BM [84,85]. In vitro, macrophages could be replaced by adherent feeder layers, however, in the absence of external forces to provide the right tension, enucleation under in vitro conditions is still a challenge. Cultures derived from ESC reached 60% enucleation, but only 4–10% in iPSC-derived erythroid cultures [38]. Detailed understanding of the mechanisms of enucleation and its stimulants/inhibitors would help design the optimal conditions for maximal enucleation: For example, modulators of intracellular vesicle trafficking, the Rho-associated protein kinase and nonmuscle myosin IIB, play essential roles not only in cytokinesis but also in enucleation of human erythroblasts [86,87]. Factors that promote the functionality of proteins involved in vesicle trafficking, such as vacuolin-1, also increase enucleation [87]. Histone deacetylases (particularly, the HDAC2 isoform), are required for chromatin condensation and enucleation in mouse fetal erythroblasts [88]. Glucocorticoids, which are strong inhibitors of HDAC2 [89], may be responsible for the poor enucleation in vitro; this could be improved by adding HDAC activators.

(E) For clinical applications, the use of animal products, e.g., serum, or feeder cells in the cultures should be avoided. A medium that includes human proteins instead of animal products that is of equal efficacy has been developed [90]. Cord blood feeder-free protocols have already been optimized in this regard [91], and the use of alternatives such as human plasma [38] or serum needs to be tested.

The use of stromal cells in the cultures, mainly to achieve maximal enucleation, introduces a poorly defined source of potentially immunogenic and/or infectious agents posing a challenge to the development of Good Manufacturing Practice (GMP) cRBC procedures. Attempts have been made to identify biochemical, such as mifepristone and plasmanate [92], to substitute the feeder layers.

(F) cRBC, especially when derived from embryonic tissues, CB, or iPSC, contain a high content of HbF. This Hb has a higher oxygen affinity than HbA, intended to facilitate oxygen delivery from the maternal to embryonic RBC through the placenta. In post-natal life, however, the higher oxygen affinity of HbF may result in lower oxygen delivery to tissues [93]. This is most probably not a health issue since individuals with hereditary persistence of fetal hemoglobin that have high HbF (as high as 100%) are largely asymptomatic [94]. Moreover, as discussed above, in patients with β-hemoglobinopathies, including β-thal, high HbF ameliorates the symptoms (for review see [63]. In principle, high HbF-containing cRBC produced from the patient’s PB progenitors or by the iPS technology from various somatic cells could be used for autologous transfusion. Moreover, the HbF content of these cRBC could be further stimulated by the addition of HbF-stimulating agents during specific phases of the culture [65].

(G) Long-term in vitro expansion of cells tends to induce karyotypic abnormalities and thus present a risk to become oncogenic. Although the cultures yield mainly mature RBC that do not contain genetic material, contaminating nucleated cells, capable of proliferation, may present a potential risk. This could be avoided by procedures (leuko-reduction plus radiation) developed to prevent allo-immunization to human leukocyte antigen (HLA) present on leukocytes and graft versus host disease after HSC transplantation.

(H) Finally, the cost of RBC production is a crucial factor. This includes the necessary sophisticated facilities, proficient staff, and expensive disposable components, especially cytokines. The volume required in static cultures was estimated to be 1000 liters per one blood unit. Regarding this issue, a simplified, inexpensive, medium, which contains eight defined components, was recently reported to expand human iPSCs [5].

Many of these issues have been already outlined) and extensive characterization of the physical properties and Hb content of RBC expanded from donors have been published [95].

## 7. Conclusions

In vitro hematopoiesis, including erythropoiesis, has made a long way since its birth some 50 years ago. The initiating cells of the cultures and the procedures employed have varied widely and the yield of cells increased significantly. The cultures have contributed to the understanding of various aspects of the basic biology of normal erythroid development and its pathology in various diseases. In thal, they were utilized for developing innovative therapeutics, such as new drugs, and methods of gene therapy, as well as potential alternative sources for RBC transfusions. Since patients, particularly patients with thal, are diverse in their clinical presentation and in their response to therapy, primary cultures derived from the patients’ PB might be used as a predictive assay for the outcome of various therapies (e.g., HbF-stimulating drugs) and thus assist in designing optimal personalized medicine. In gene therapy, various selection protocols for gene-modified cells may be applied in cultures of HSC or iPSC, prior to transplanting them back to the patient. In vitro production of RBC, particularly those with rare phenotypes, for transfusion is another fast progressing area. A major obstacle to the wide application of cultures in clinical laboratories is the poor proficiency of non-specialized labs. Developing standard protocols and reagents as well as establishing central cellular bio-banks and specialized processing labs could be helpful.
